# Majorization, Csiszár divergence and Zipf-Mandelbrot law

**DOI:** 10.1186/s13660-017-1472-2

**Published:** 2017-08-24

**Authors:** Naveed Latif, Ðilda Pečarić, Josip Pečarić

**Affiliations:** 1 Department of Social Studies, Jubail Industrial College, Jubail, 31961 Kingdom of Saudi Arabia; 2 Catholic University of Croatia, Ilica 242, Zagreb, 10000 Croatia; 3 Faculty of Textile Technology Zagreb, University of Zagreb, Prilaz Baruna Filipovića 30, Zagreb, 10000 Croatia

**Keywords:** 94A15, 94A17, 26A51, 26D15, majorization inequality, Csiszár *f*-divergence, Shannon entropy, Kullback-Leibler divergence, Zipf-Mandelbrot law

## Abstract

In this paper we show how the Shannon entropy is connected to the theory of majorization. They are both linked to the measure of disorder in a system. However, the theory of majorization usually gives stronger criteria than the entropic inequalities. We give some generalized results for majorization inequality using Csiszár *f*-divergence. This divergence, applied to some special convex functions, reduces the results for majorization inequality in the form of Shannon entropy and the Kullback-Leibler divergence. We give several applications by using the Zipf-Mandelbrot law.

## Introduction and preliminaries

Well over a century ago measures were derived for assessing the distance between two models of probability distributions. Most relevant is Boltzmann’s [1] concept of generalized entropy in physics and thermodynamics (see Akaike [2] for a brief review). Shannon [3] employed entropy in his famous treatise on communication theory. Kullback-Leibler [4] derived an information measure that happened to be the negative of Boltzmann’s entropy, now referred to as the Kullback-Leibler (K-L) distance. The motivation for the Kullback-Leibler work was to provide a rigorous definition of information in relation to Fisher’s sufficient statistics. The K-L distance has also been called the K-L discrepancy, divergence, information and number. These terms are synonyms; we use the term ’distance’ in the material to follow.

A fundamental result related to the notion of the Shannon entropy is the following inequality (see [5]):

1 $$\begin{aligned} \sum_{i=1}^{n} p_{i} \log\frac{1}{p_{i}} \leq\sum_{i=1}^{n} p_{i} \log\frac{1}{q_{i}}, \end{aligned}$$

for all positive real numbers $p_{i}$ and $q_{i}$ with

2 $$\begin{aligned} \sum_{i=1}^{n} p_{i} = \sum_{i=1}^{n} q_{i}. \end{aligned}$$

Here, ‘log’ denotes the logarithmic function taken to a fixed base $b > 1$. Equality holds in (1) if $q_{i}=p_{i}$ for all i. For details, see [6], p.635-650. This result, sometimes called the fundamental lemma of information theory, has extensive applications (see for example [7]).

Matić *et al.* [5, 8, 9] and [10] continuously worked on Shannon’s inequality and related inequalities in the probability distribution and information science. They studied and discussed in [5, 10] several aspects of Shannon’s inequality in discrete as well as in integral forms, by presenting upper estimates of the difference between its two sides. Applications to the bounds in information theory were also given.

Now we introduce the main mathematical theory explored in the presented work, the theory of majorization. It is a powerful and elegant mathematical tool which can be applied to a wide variety of problems as well as in quantum mechanics. The theory of majorization is closely related to the notions of randomness and disorder. It indeed allows us to compare two probability distributions in order to know which one is more random. Let us now give the most general definition of majorization.

For fixed $n\geq2$ let

$$ \textbf{x} = (x_{1}, \ldots, x_{n} ), \qquad \textbf{y} = (y_{1}, \ldots, y_{n} ) $$

denote two real *n*-tuples. Let

$$\begin{aligned} &x_{[1]} \geq x_{[2]} \geq\cdots\geq x_{[n]},\qquad y_{[1]} \geq y_{[2]} \geq\cdots\geq y_{[n]}, \\ &x_{(1)} \leq x_{(2)} \leq\cdots\leq x_{(n)},\qquad y_{(1)} \leq y_{(2)} \leq\cdots\leq y_{(n)} \end{aligned}$$

denote their ordered components.

The following definition is given in [11], p.319.

*Majorization*: Let $\textbf{x} = (x_{1}, \ldots, x_{n} ), \textbf{y} = (y_{1}, \ldots, y_{n} )$ be *n*-tuples of real numbers. Then we say that **y** is majorized by **x** or that **x** majorizes **y**, in symbol, $\mathbf{x}\succ \mathbf{y}$, if we have

3 $$ \sum_{i=1}^{j} y_{[i]}\leq\sum_{i=1}^{j} x_{[i]}, $$

for $j= 1, 2, \ldots, n-1$, and

4 $$ \sum_{i=1}^{n} x_{[i]} = \sum_{i=1}^{n} y_{[i]}. $$

Note that (3) is equivalent to

$$ \sum_{i=n-j+1}^{n} y_{(i)} \leq\sum _{i=n-j+1}^{n} x_{(i)}, $$

for $j=1, 2, \ldots, n-1$.

The following theorem, called the classical majorization theorem, is given in the monograph by Marshall *et al.* [12], p.11 (see also [11], p.320):

### Theorem 1

Classical majorization theorem

*Let* $\textbf{x}= (x_{1}, \ldots, x_{n} ), \textbf{y} = (y_{1}, \ldots, y_{n} )$ *be two real* *n*-*tuples such that* $x_{i}$, $y_{i}$ $\in J \subset\mathbb{R}$ *for* $i=1, \ldots, n$. *Then* **x** *majorizes* **y** *if and only if for every continuous convex function* $f:J \rightarrow\mathbb{R}$, *the following inequality holds*:

5 $$ \sum_{i=1}^{n} f (y_{i} ) \leq\sum_{i=1}^{n} f (x_{i} ). $$

The following theorem is a generalization of Theorem 1, known as weighted majorization theorem, and was proved by Fuchs in [13] (see also [11], p.323):

### Theorem 2

Weighted majorization theorem

*Let* $\mathbf{x}= (x_{1}, \ldots, x_{n} ), \mathbf{y} = (y_{1}, \ldots, y_{n} )$ *be two decreasing real* *n*-*tuples such that* $x_{i}$, $y_{i}$ ∈*J* *for* $i=1, \ldots, n$. *Let* $\mathbf{w}=(w_{1}, \ldots, w_{n})$ *be a real* *n*-*tuple such that*

6 $$ \sum_{i=1}^{j} w_{i} y_{i} \leq\sum_{i=1}^{j} w_{i} x_{i}, $$

*for* $j=1,2, \ldots, n-1$, *and*

7 $$ \sum_{i=1}^{n} w_{i} y_{i} = \sum_{i=1}^{n} w_{i} x_{i}. $$

*Then*, *for every continuous convex function* $f:J \rightarrow\mathbb {R}$, *we have the following inequality*:

8 $$ \sum_{i=1}^{n} w_{i} f (y_{i} ) \leq\sum_{i=1}^{n} w_{i} f (x_{i} ). $$

The following theorem is valid ([14], p.32).

### Theorem 3

*Let* $f:J\rightarrow R$ *be a continuous convex function on an interval* *J*, **w** *be a positive* *n*-*tuple*, *and* **x**, **y** $\in J^{n}$ *satisfy*

9 $$ \sum_{i=1}^{k} w_{i} y_{i} \leq\sum_{i=1}^{k} w_{i} x_{i} \quad \textit{for } k=1, \ldots, n-1, $$

*and*

10 $$ \sum_{i=1}^{n} w_{i} y_{i} = \sum_{i=1}^{n} w_{i} x_{i}. $$

- *If* **y** *is a decreasing* *n*-*tuple*, *then*
  11 $$ \sum_{i=1}^{n} w_{i} f (y_{i} ) \leq\sum_{i=1}^{n} w_{i} f (x_{i} ). $$
- *If* **x** *is an increasing* *n*-*tuple*, *then*
  12 $$ \sum_{i=1}^{n} w_{i} f (x_{i} ) \leq\sum_{i=1}^{n} w_{i} f (y_{i} ). $$

*If* *f* *is strictly convex and* $\mathbf{x} \neq\mathbf{y}$, *then* (11) *and* (12) *are strict*.

Matić *et al.* [5, 10] considered a discrete-valued random variable X with finite range $\{x_{i}\}_{i=1}^{r}$. Assume $p_{i}=P\{X=x_{i}\}$. The b-entropy of X is defined by

13 $$\begin{aligned} H_{b}(X):=\sum_{i=1}^{r} p_{i} \log(1/p_{i}). \end{aligned}$$

In [5], they proved that

14 $$\begin{aligned} H_{b}(X)\leq\log r, \end{aligned}$$

which shows that the entropy function $H_{b}(X)$ reaches its maximum value on the discrete uniform probability distribution.

They introduced the idea by giving the general setting of the above inequality by using the classical majorization theorem for the function $f(x)= x \log x$, which is convex and continuous on $\mathbf{R}_{+}$. Suppose *X* and *Y* are discrete random variables with finite ranges and probability distributions $\mathbf{p}=\{p_{i}\}_{i=1}^{r}$ and $\mathbf{q}= \{q_{i}\}_{i=1}^{r}$ $(\sum_{i=1}^{r} p_{i}=\sum_{i=1}^{r} q_{i}= 1 )$, such that $\mathbf{p}\succ\mathbf{q}$. Then by the majorization theorem

15 $$\begin{aligned} H_{b}(X) \leq H_{b} (Y). \end{aligned}$$

By substituting $\mathbf{p} > (1/r, \ldots, 1/r )$ we get (14).

It is generally common to take log with a base of 2 in the introduced notions, but in our investigations this is not essential.

In Section 2, we present our main generalized results obtained from majorization inequality by using Csiszár *f*-divergence and then obtain corollaries in the form of Shannon entropy and the K-L distance. In Section 3, we give several applications using the Zipf-Mandelbrot law.

## Csiszár *f*-divergence for majorization

Csiszár introduced in [15] and then discussed in [16] the following notion.

### Definition 1

Let $f: \mathbb{R}_{+} \rightarrow\mathbb{R}_{+}$ be a convex function, and let $\mathbf{p}:= (p_{1}, \ldots, p_{n} )$ and $\mathbf{q}:= (q_{1}, \ldots, q_{n} )$ be positive probability distributions. The *f*-divergence functional is

$$\begin{aligned} I_{f} ( \mathbf{p}, \mathbf{q} ):= \sum_{i=1}^{n} q_{i} f \biggl( \frac{p_{i}}{q_{i}} \biggr). \end{aligned}$$

It is possible to use non-negative probability distributions in the *f*-divergence functional, by defining

$$\begin{aligned} f(0):=\lim_{t \rightarrow0^{+}} f(t);\qquad 0f \biggl(\frac{0}{0} \biggr):=0;\qquad 0f \biggl(\frac{a}{0} \biggr):=\lim_{t\rightarrow0^{+}} t f \biggl( \frac{a}{t} \biggr),\quad a> 0. \end{aligned}$$

Horváth *et al.* [17], p.3, considered functionality based on the previous definition.

### Definition 2

Let $J\subset\mathbb{R}$ be an interval, and let $f:J\rightarrow \mathbb{R}$ be a function. Let $\mathbf{p}:= (p_{1}, \ldots, p_{n} ) \in\mathbb{R}^{n}$, and $\mathbf{q}:= (q_{1}, \ldots, q_{n} ) \in \,]0, \infty [^{n}$ be such that

16 $$\begin{aligned} \frac{p_{i}}{q_{i}} \in J,\quad i=1, \ldots, n. \end{aligned}$$

Then we denote

$$\begin{aligned} \hat{I}_{f} ( \mathbf{p}, \mathbf{q} ):= \sum _{i=1}^{n} q_{i} f \biggl( \frac{p_{i}}{q_{i}} \biggr). \end{aligned}$$

Motivated by the ideas in [5] and [10], in this paper we study and discuss the majorization results in the form of divergences and entropies. The following theorem is a generalization of the result given in [5], *i.e.*, (15).

Assume **p** and **q** to be *n*-tuples, then we define

$$\begin{aligned} \frac{\mathbf{p}}{\mathbf{q}}:= \biggl(\frac{p_{1}}{q_{1}}, \frac {p_{2}}{q_{2}}, \ldots, \frac{p_{n}}{q_{n}} \biggr). \end{aligned}$$

The following theorem is the connection between Csiszár *f*-divergence and weighted majorization inequality as one sequence is monotonic.

### Theorem 4

*Assume* $J\subset\mathbf{\mathbb{R}}$ *to be an interval*, $f: J\rightarrow\mathbb{R}$ *to be a continuous convex function*, $p_{i}$, $r_{i}$ $(i=1, \ldots, n)$ *to be real numbers and* $q_{i}$ ($i=1, \ldots, n$) *to be positive real numbers*, *such that*

17 $$ \sum_{i=1}^{k} r_{i} \leq\sum_{i=1}^{k} p_{i}, \quad \textit{for } k=1, \ldots, n-1, $$

*and*

18 $$ \sum_{i=1}^{n} r_{i} = \sum_{i=1}^{n} p_{i}, $$

*with* $\frac{p_{i}}{q_{i}}, \frac{r_{i}}{q_{i}} \in J$ ($i=1,\ldots, n$).

- *If* $\frac{\mathbf{r}}{\mathbf{q}}$ *is decreasing*, *then*
  19 $$ \hat{I}_{f} (\mathbf{r}, \mathbf{q} ) \leq \hat {I}_{f} (\mathbf{p}, \mathbf{q} ). $$
- *If* $\frac{\mathbf{p}}{\mathbf{q}}$ *is increasing*, *then*
  20 $$ \hat{I}_{f} (\mathbf{r}, \mathbf{q} ) \geq \hat {I}_{f} (\mathbf{p}, \mathbf{q} ). $$
  *If* *f* *is a continuous concave function*, *then the reverse inequalities hold in* (19) *and* (20).

### Proof

(a): We use Theorem 3(a) with substitutions $x_{i}:=\frac {p_{i}}{q_{i}}$, $y_{i}:=\frac{r_{i}}{q_{i}}$, $w_{i}:= q_{i}$ and $q_{i} >0$ ($i=1, \ldots, n$). Then we get (19).

We can prove part (b) with similar substitutions in Theorem 3(b). □

### Theorem 5

*Assume* $J\subset\mathbf{\mathbb{R}}$ *to be an interval*, $g: J\rightarrow\mathbb{R}$ *to be a function*, *such that* $x\rightarrow x g(x)$ $(x \in J)$ *to be a continuous convex function*, $p_{i}$ *and* $r_{i}$ $(i=1, \ldots, n)$ *to be real numbers and* $q_{i}$ ($i=1, \ldots, n$) *to be positive real numbers satisfying* (17) *and* (18) *with*

$$\begin{aligned} \frac{p_{i}}{q_{i}}, \frac{r_{i}}{q_{i}} \in J\quad (i=1,\ldots, n). \end{aligned}$$

- *If* $\frac{\mathbf{r}}{\mathbf{q}}$ *is decreasing*, *then*
  21 $$ \hat{I}_{g} (\mathbf{r}, \mathbf{q} ):=\sum _{i=1}^{n} r_{i} g \biggl( \frac{r_{i}}{q_{i}} \biggr) \leq \hat{I}_{g} (\mathbf{p}, \mathbf{q} ). $$
- *If* $\frac{\mathbf{p}}{\mathbf{q}}$ *is increasing*, *then*
  22 $$ \hat{I}_{g} (\mathbf{r}, \mathbf{q} ) \geq \hat {I}_{g} (\mathbf{p}, \mathbf{q} ). $$
  *If* $xg(x)$ *is a continuous concave function*, *then the reverse inequalities hold in* (21) *and* (22).

### Proof

(a): We use Theorem 3(a) with substitutions $x_{i}=\frac {p_{i}}{q_{i}}$, $y_{i}=\frac{r_{i}}{q_{i}}$, $w_{i}= q_{i}$ as $q_{i} >0$ ($i=1, \ldots, n$), and $f(x):= xg(x)$. Then we get (21).

We can prove part (b) with similar substitutions in Theorem 3(b) for $f(x):= xg(x)$. □

The theory of majorization and the notion of entropic measure of disorder are closely related. Based on this fact, the aim of this paper is to look for majorization relations with the connection to entropic inequalities. This was interesting to do for two main reasons. The first one is the fact that the majorization relations are usually stronger than the entropic inequalities, in the sense that they imply these entropic inequalities, but the converse is not true. The second reason is the fact that, when we dispose of majorization relations between two different quantum states, we know that we can transform one of the states into the other using some unitary transformation. The concept of entropy alone would not allow us to prove such a property.

The Shannon entropy was introduced in the field of classical information. There are two ways of viewing the Shannon entropy. Suppose we have a random variable *X*, and we learn its value. In one point of view, the Shannon entropy quantifies the amount of information as regards the value of *X* (after measurement). In another point of view, the Shannon entropy tells us the amount of uncertainty about the variable of *X* before we learn its value (before measurement).

We mention two special cases of the previous result.

The first case corresponds to the entropy of a discrete probability distribution.

### Definition 3

The Shannon entropy of a positive probability distribution $\mathbf {p}:= (p_{1}, \ldots, p_{n} )$ is defined by

23 $$\begin{aligned} H(p):= - \sum_{i=1}^{n} p_{i} \log p_{i}. \end{aligned}$$

Note that there is no problem with the definition in the case of a zero probability, since

24 $$\begin{aligned} \lim_{x\rightarrow0} x \log x=0. \end{aligned}$$

### Corollary 1

*Assume* $p_{i}$, $r_{i}$ *and* $q_{i}$ $(i=1, \ldots, n)$ *to be positive real numbers satisfying* (17) *and* (18) *with*

$$\begin{aligned} \frac{p_{i}}{q_{i}}, \frac{r_{i}}{q_{i}} \in J\quad (i=1,\ldots, n). \end{aligned}$$

- *If* $\frac{\mathbf{r}}{\mathbf{q}}$ *is a decreasing* *n*-*tuple and the base of* log *is greater than* 1, *then the following estimates for the Shannon entropy of* **q** *hold*:
  25 $$ \sum_{i=1}^{n} q_{i} \log \biggl(\frac{r_{i}}{q_{i}} \biggr) \geq H(\mathbf{q}). $$
  *If the base of* log *is in between* 0 *and* 1, *then the reverse inequality holds in* (25).
- *If* $\frac{\mathbf{p}}{\mathbf{q}}$ *is an increasing* *n*-*tuple and the base of* log *is greater than* 1, *then the following estimates for the Shannon entropy of* **q** *hold*:
  26 $$ H(\mathbf{q}) \leq \sum_{i=1}^{n} q_{i}\log \biggl(\frac{p_{i}}{q_{i}} \biggr). $$
  *If the base of* log *is in between* 0 *and* 1, *then the reverse inequality holds in* (26).

### Proof

(a): Substitute $f(x):= \log x$ and $p_{i}=1$ ($i=1, \ldots, n$) in Theorem 4(a). Then we get (25).

We can prove the part (b) with similar substitutions for $r_{i}=1$ ($i=1, \ldots, n$). □

### Corollary 2

*Assume* $p_{i}$ *and* $r_{i}$ $(i=1, \ldots, n)$ *to be positive real numbers satisfying* (17) *and* (18).

- *If* **r** *is a decreasing* *n*-*tuple and the base of* log *is greater than* 1, *then for the connection between the Shannon entropies of* **p** *and* **r**
  27 $$ H(\mathbf{r}) \geq H(\mathbf{p}). $$
  *If the base of* log *is in between* 0 *and* 1, *then the reverse inequality holds in* (27).
- *If* **p** *is an increasing* *n*-*tuple and the base of* log *is greater than* 1, *then for the connection between Shannon entropies of* **p** *and* **r**
  28 $$ H(\mathbf{r}) \leq H(\mathbf{p}). $$
  *If the base of* log *is in between* 0 *and* 1, *then the reverse inequality holds in* (28).

### Proof

(a): Substitute $g(x):= \log x$ and $q_{i}=1$ ($i=1, \ldots, n$) in Theorem 5(a). Then we get (27).

We can prove part (b) with similar substitutions. □

The second case corresponds to the relative entropy or the K-L distance between two probability distributions.

### Definition 4

The K-L distance between the positive probability distributions $\mathbf{p}:= (p_{1}, \ldots, p_{n} )$ and $\mathbf{q}:= (q_{1}, \ldots, q_{n} )$ is defined by

$$\begin{aligned} L (\mathbf{p},\mathbf{q} ):= \sum_{i=1}^{n} p_{i} \log \biggl(\frac{p_{i}}{q_{i}} \biggr). \end{aligned}$$

### Corollary 3

*Assume* $J\subset\mathbf{\mathbb{R}}$ *to be an interval*, *and* $p_{i}$, $r_{i}$ *and* $q_{i}$ ($i=1, \ldots, n$) *to be positive real numbers satisfying* (17) *and* (18) *with*

$$\begin{aligned} \frac{p_{i}}{q_{i}}, \frac{r_{i}}{q_{i}} \in J\quad (i=1,\ldots, n). \end{aligned}$$

- *If* $\frac{\mathbf{r}}{\mathbf{q}}$ *is a decreasing* *n*-*tuple and the base of* log *is greater than* 1, *then*
  29 $$ \sum_{i=1}^{n} q_{i} \log \biggl(\frac{r_{i}}{q_{i}} \biggr) \geq \sum _{i=1}^{n} q_{i} \log \biggl( \frac{p_{i}}{q_{i}} \biggr). $$
  *If the base of* log *is in between* 0 *and* 1, *then the reverse inequality holds in* (29).
- *If* $\frac{\mathbf{p}}{\mathbf{q}}$ *is an increasing* *n*-*tuple and the base of* log *is greater than* 1, *then*
  30 $$ \sum_{i=1}^{n} q_{i} \log \biggl(\frac{r_{i}}{q_{i}} \biggr) \leq \sum _{i=1}^{n} q_{i} \log \biggl( \frac{p_{i}}{q_{i}} \biggr). $$
  *If the base of* log *is in between* 0 *and* 1, *then the reverse inequality holds in* (30).

### Proof

(a): Substitute $f(x):= \log x$ in Theorem 4(a). Then we get (29).

We can prove part (b) with substitution $f(x):= \log x$ in Theorem 4(b). □

### Corollary 4

*Let* $J\subset\mathbf{\mathbb{R}}$ *be an interval and assume* $p_{i}$, $r_{i}$ *and* $q_{i}$ ($i=1, \ldots, n$) *be positive real numbers satisfying* (17) *and* (18) *with*

$$\begin{aligned} \frac{p_{i}}{q_{i}}, \frac{r_{i}}{q_{i}} \in J \quad (i=1,\ldots, n). \end{aligned}$$

- *If* $\frac{\mathbf{r}}{\mathbf{q}}$ *is a decreasing* *n*-*tuple and the base of* log *is greater than* 1, *then the following comparison inequality between K*-*L distance of* $(\mathbf {r},\mathbf{q})$ *and* $(\mathbf{p}, \mathbf{q})$ *holds*:
  31 $$ L (\mathbf{r},\mathbf{q} ):=\sum_{i=1}^{n} r_{i} \log \biggl(\frac{r_{i}}{q_{i}} \biggr) \leq L (\mathbf{p}, \mathbf {q} ):= \sum_{i=1}^{n} p_{i} \log \biggl(\frac{p_{i}}{q_{i}} \biggr). $$
  *If the base of* log *is in between* 0 *and* 1, *then the reverse inequality holds in* (31).
- *If* $\frac{\mathbf{p}}{\mathbf{q}}$ *is an increasing* *n*-*tuple and the base of* log *is greater than* 1, *then the following comparison inequality between K*-*L distance of* $(\mathbf {r},\mathbf{q})$ *and* $(\mathbf{p}, \mathbf{q})$ *holds*:
  32 $$ \sum_{i=1}^{n} r_{i} \log \biggl(\frac{r_{i}}{q_{i}} \biggr) \geq \sum _{i=1}^{n} p_{i} \log \biggl( \frac{p_{i}}{q_{i}} \biggr). $$
  *If the base of* log *is in between* 0 *and* 1, *then the reverse inequality holds in* (32).

### Proof

(a): Substitute $g(x):= \log x$ in Theorem 5(a). Then we get (31).

We can prove part (b) with substitution $g(x):= \log x$ in Theorem 5(b). □

### Remark 1

We give the above results when one sequence is monotone by using Theorem 3, but we can give all the above results when both sequences are monotone by using the weighted majorization theorem, Theorem 2, for $w_{i}>0$ $(i=1, \ldots, n)$.

## Applications to the Zipf-Mandelbrot entropy

The term Zipfian distribution refers to a distribution of probabilities of occurrence that follows Zipf’s law. Zipf’s law is an experimental law, not a theoretical one; *i.e.* it describes an occurrence rather than predicting it from some kind of theory: the observation that, in many natural and man-made phenomena, the probability of occurrence of many random items starts high and tapers off. Thus, a few occur very often while many others occur rarely. The formal definition of this law is $\mathbf{P_{n}}= 1/\mathbf{n^{a}}$, where **Pn** is the frequency of occurrence of the **n**th ranked item and **a** is close to 1.

Converted to language, this means that the rank of a word (in terms of its frequency) is approximately inversely proportional to its actual frequency, and so produces a hyperbolic distribution. To put Zipf’s law in another way (see [18, 19]): $fr=C$, where *r*= the rank of a word, *f*= the frequency of occurrence of that word, and *C*= a constant (the value of which depends on the subject under consideration). Essentially this shows an inverse proportional relationship between a word’s frequency and its frequency rank. Zipf called this curve the ‘standard curve’. Texts from natural languages do not, of course, behave with such absolute mathematical precision. They can not, because, for one thing, any curve representing empirical data from large texts will be a stepped graph, since many non-high-frequency words will share the same frequency. But the overall consensus is that texts match the standard curve significantly well. Li [20] writes ‘this distribution, also called Zipf’s law, has been checked for accuracy for the standard corpus of the present-day English [Kućera and Francis] with very good results.’ See Miller [21] for a concise summary of the match between actual data and the standard curve.

Zipf also studied the relationship between the frequency of occurrence of a word and its length. In The Psycho-Biology of Language, he stated that ‘it seems reasonably clear that shorter words are distinctly more favored in language than longer words.’

Apart from the use of this law in information science and linguistics, Zipf’s law is used in economics. This distribution in economics is known as Pareto’s law, which analyzes the distribution of the wealthiest members of the community [22], p.125. These two laws are the same in the mathematical sense, but they are applied in different contexts [23], p.294. The same type of distribution that we have in Zipf’s and Pareto’s law, also known as the power law, can also be found in other scientific disciplines, such as physics, biology, earth and planetary sciences, computer science, demography and the social sciences [24].

Benoit Mandelbrot in [25] gave a generalization of Zipf’s law, now known as the Zipf-Mandelbrot law, which gave an improvement in the account for the low-rank words in a corpus where $k < 100$ [26]:

$$\begin{aligned} f(k)= \frac{C}{(k+q)^{s}}, \end{aligned}$$

when $q=0$, we get Zipf’s law.

For $n \in\mathbb{N}$, $q\geq0$, $s>0$, $k \in\{1, 2, \ldots, n\} $, in a more clear form, the Zipf-Mandelbrot law (probability mass function) is defined with

33 $$\begin{aligned} f (k, n, q, s ):= \frac{1/(k+q)^{s}}{H_{n, q, s}}, \end{aligned}$$

where

34 $$\begin{aligned} H_{n, q, s}:= \sum_{i=1}^{n} \frac{1}{(i+q)^{s}}, \end{aligned}$$

$n \in\mathbb{N}$, $q\geq0$, $s>0$, $k \in\{1, 2, \ldots, n\}$.

Application of the Zipf-Mandelbrot law can also be found in linguistics [26], information sciences [19, 23] and ecological field studies [27].

In probability theory and statistics, the cumulative distribution function (CDF) of a real-valued random variable *X*, or just distribution function of *X*, evaluated at *x*, is the probability that *X* will take a value less than or equal to *x* and we often denote by CDF the following ratio:

35 $$\begin{aligned} \mathrm{CDF}:=\frac{H_{k, t, s}}{H_{n, t, s}}. \end{aligned}$$

The cumulative distribution function is an important application of majorization.

In the case of a continuous distribution, it gives the area under the probability distribution functions, also used to specify the distribution of multivariable random variables.

There are various applications of CDF. For example, in learning to rank, the CDF arises naturally as a probability measure over inequality events of the type $\{X \leq x\}$. The joint CDF lends itself to problems that are easily described in terms of inequality events in which statistical dependence relationships also exist among events. Examples of this type of problem include web search and document retrieval [28–31], predicting rating of movies [32] or predicting multiplayer game outcomes with a team structure [33]. In contrast to the canonical problems of classification or regression, in learning to rank we are required to learn some mapping from inputs to inter-dependent output variables, so that we may wish to model both stochastic orderings of variable states and statistical dependence relationships between variables.

In the following application, we use two of the Zipf-Mandelbrot laws for different parameters.

### Application 1

Assume **p** and **r** to be the Zipf-Mandelbrot laws with parameters $n \in\{ 1, 2, \ldots\}$, $t_{1}, t_{2} \geq0$ and $s_{1}, s_{2} > 0$, respectively, satisfying

36 $$\begin{aligned} \frac{H_{k, t_{2}, s_{2}}}{H_{n, t_{2}, s_{2}}} \leq\frac{H_{k, t_{1}, s_{1}}}{H_{n, t_{1}, s_{1}}},\quad k=1, \ldots, n-1, \end{aligned}$$

and also let $q_{i} >0$ $(i=1, 2, \ldots, n)$.

- If $\frac{(i+t_{2})^{s_{2}}}{(i+1+t_{2})^{s_{2}}} \leq \frac{q_{i+1}}{q_{i}}$ $(i=1, \ldots, n)$ and the base of log is greater than 1, then
  37 $$\begin{aligned} &\sum_{i=1}^{n} \frac{1}{(i+t_{2})^{s_{2}} H_{n, t_{2}, s_{2}}} \log \biggl(\frac{1}{q_{i} (i+t_{2})^{s_{2}} H_{n, t_{2}, s_{2}}} \biggr) \\ &\quad \leq \sum_{i=1}^{n} \frac{1}{(i+t_{1})^{s_{1}} H_{n, t_{1}, s_{1}}} \log \biggl(\frac{1}{q_{i} (i+t_{1})^{s_{1}} H_{n, t_{1}, s_{1}}} \biggr). \end{aligned}$$
  If the base of log is in between 0 and 1, then the reverse inequality holds in (37).
- If $\frac{(i+t_{1})^{s_{1}}}{(i+1+t_{1})^{s_{1}}} \geq \frac{q_{i+1}}{q_{i}}$ $(i=1, \ldots, n)$ and the base of log is greater than 1, then
  38 $$\begin{aligned} &\sum_{i=1}^{n} \frac{1}{(i+t_{2})^{s_{2}} H_{n, t_{2}, s_{2}}} \log \biggl(\frac{1}{q_{i} (i+t_{2})^{s_{2}} H_{n, t_{2}, s_{2}}} \biggr) \\ &\quad \geq\sum_{i=1}^{n} \frac{1}{(i+t_{1})^{s_{1}} H_{n, t_{1}, s_{1}}} \log \biggl(\frac{1}{q_{i} (i+t_{1})^{s_{1}} H_{n, t_{1}, s_{1}}} \biggr). \end{aligned}$$
  If the base of log is in between 0 and 1, then the reverse inequality holds in (38).

### Proof

(a) Assume $p_{i}:= \frac{1}{(i+t_{1})^{s_{1}} H_{n, t_{1}, s_{1}}}$ and $r_{i}:= \frac{1}{(i+t_{2})^{s_{2}} H_{n, t_{2}, s_{2}}}$, then

$$\begin{aligned} \sum_{i=1}^{k} p_{i} := \sum _{i=1}^{k} \frac {1}{(i+t_{1})^{s_{1}}H_{n, t_{1}, s_{1}}} = \frac{1}{H_{n, t_{1}, s_{1}}} \sum_{i=1}^{k} \frac{1}{(i+t_{1})^{s_{1}}} = \frac{H_{k, t_{1}, s_{1}}}{H_{n, t_{1}, s_{1}}},\quad k=1, \ldots, n-1. \end{aligned}$$

Similarly, $\sum_{i=1}^{k} r_{i} := \frac{H_{k, t_{2}, s_{2}}}{H_{n, t_{2}, s_{2}}}, k=1, \ldots, n-1$.

This implies that

$$\begin{aligned} \sum_{i=1}^{k} r_{i} \leq\sum _{i=1}^{k} p_{i} \quad \Leftrightarrow\quad \frac {H_{k, t_{2}, s_{2}}}{H_{n, t_{2}, s_{2}}} \leq\frac{H_{k, t_{1}, s_{1}}}{H_{n, t_{1}, s_{1}}},\quad k=1, \ldots, n-1. \end{aligned}$$

We can easily check that $\frac{1}{(i+t_{1})^{s_{1}} H_{n, t_{1}, s_{1}}}$ is decreasing over $i=1, \ldots, n$ and similarly $r_{i}$ too. Now, we investigate the behavior of $\frac{\mathbf{r}}{\mathbf{q}}$ for $q_{i}>0$ $(i=1, 2, \ldots, n)$; take

$$\begin{aligned} &\frac{r_{i}}{q_{i}}= \frac{1}{q_{i}(i+t_{2})^{s_{2}}H_{n, t_{2}, s_{2}}} \quad \mbox{and}\quad \frac{r_{i+1}}{q_{i+1}}= \frac {1}{q_{i+1}(i+1+t_{2})^{s_{2}} H_{n, t_{2}, s_{2}}}, \\ &\frac{r_{i+1}}{q_{i+1}} - \frac{r_{i}}{q_{i}} = \frac{1}{H_{n, t_{2}, s_{2}}} \biggl[ \frac{1}{q_{i+1} (i+1+t_{2})^{s_{2}}} - \frac{1}{q_{i} (i+t_{2})^{s_{2}}} \biggr] \leq0 \\ &\quad \Leftrightarrow\quad \frac{(i+t_{2})^{s_{2}}}{(i+1+t_{2})^{s_{2}}} \leq \frac{q_{i+1}}{q_{i}}, \end{aligned}$$

which shows that $\frac{\mathbf{r}}{\mathbf{q}}$ is decreasing. So all the assumptions of Corollary 4(a) are true. Then by using (31) we get (37).

(b) If we switch the role of $r_{i}$ into $p_{i}$, then by using (32) in Corollary 4(b) we get (38). □

The following application is a special case of the above result.

### Application 2

Assume **p** and **r** to be the Zipf-Mandelbrot laws with parameters $n \in\{ 1, 2, \ldots\}$, $t_{1}, t_{2} \geq0$ and $s_{1}, s_{2} > 0$, respectively, satisfying (36).

If the base of log is greater than 1, then

39 $$\begin{aligned} &\sum_{i=1}^{n} \frac{1}{(i+t_{2})^{s_{2}} H_{n, t_{2}, s_{2}}} \log \biggl(\frac{1}{ (i+t_{2})^{s_{2}} H_{n, t_{2}, s_{2}}} \biggr) \\ &\quad \leq\sum_{i=1}^{n} \frac{1}{(i+t_{1})^{s_{1}} H_{n, t_{1}, s_{1}}} \log \biggl(\frac{1}{ (i+t_{1})^{s_{1}} H_{n, t_{1}, s_{1}}} \biggr). \end{aligned}$$

If the base of log is in between 0 and 1, then the reverse inequality holds in (39).

### Proof

Substitute $q_{i}:=1$ $(i=1, 2, \ldots, n)$ in (37); we get (39). □

### Application 3

Assume **p** and **r** to be the Zipf-Mandelbrot laws with parameters $n \in\{ 1, 2, \ldots\}$, $t_{1}, t_{2} \geq0$ and $s_{1}, s_{2} > 0$, respectively, satisfying (36) and also let $q_{i} >0$ $(i=1, 2, \ldots, n)$.

- If $\frac{(i+t_{2})^{s_{2}}}{(i+1+t_{2})^{s_{2}}} \leq \frac{q_{i+1}}{q_{i}}$ $(i=1, \ldots, n)$ and the base of log is greater than 1, then
  40 $$ \sum_{i=1}^{n} q_{i} \log \biggl(\frac{1}{q_{i} (i+t_{2})^{s_{2}} H_{n, t_{2}, s_{2}}} \biggr) \geq\sum _{i=1}^{n} q_{i} \log \biggl( \frac{1}{q_{i} (i+t_{1})^{s_{1}} H_{1, t_{1}, s_{1}}} \biggr). $$
  If the base of log is in between 0 and 1, then the reverse inequality holds in (40).
- If $\frac{(i+t_{1})^{s_{1}}}{(i+1+t_{1})^{s_{1}}} \geq \frac{q_{i+1}}{q_{i}}$ $(i=1, \ldots, n)$ and the base of log is greater than 1, then
  41 $$ \sum_{i=1}^{n} q_{i} \log \biggl(\frac{1}{q_{i} (i+t_{2})^{s_{2}} H_{n, t_{2}, s_{2}}} \biggr) \leq\sum _{i=1}^{n} q_{i} \log \biggl( \frac{1}{q_{i} (i+t_{1})^{s_{1}} H_{1, t_{1}, s_{1}}} \biggr). $$
  If the base of log is in between 0 and 1, then the reverse inequality holds in (41).

### Proof

We can prove by a similar method as given in Application 1 with substitutions $p_{i}:= \frac{1}{(i+t_{1})^{s_{1}}H_{n, t_{1}, s_{1}}}$ and $r_{i}:= \frac{1}{(i+t_{2})^{s_{2}}H_{n, t_{2}, s_{2}}}$ in Corollary 3 instead of Corollary 4, to get the required results. □

The following result is a special case of Application 3.

### Application 4

Assume **p** and **r** to be the Zipf-Mandelbrot laws with parameters $n \in\{ 1, 2, \ldots\}$, $t_{1}, t_{2} \geq0$ and $s_{1}, s_{2} > 0$, respectively, satisfying (36). If the base of log is greater than 1, then

42 $$ \sum_{i=1}^{n} \log \biggl( \frac{1}{ (i+t_{2})^{s_{2}} H_{n, t_{2}, s_{2}}} \biggr) \geq\sum_{i=1}^{n} \log \biggl(\frac {1}{(i+t_{1})^{s_{1}} H_{1, t_{1}, s_{1}}} \biggr). $$

If the base of log is in between 0 and 1, then the reverse inequality holds in (42).

### Proof

Substitute $q_{i}:=1$ $(i=1, 2, \ldots, n)$ in (40); we get (42). □

### Application 5

Assume **p** and **r** to be the Zipf-Mandelbrot laws with parameters $n \in\{ 1, 2, \ldots\}$, $t_{1}, t_{2} \geq0$ and $s_{1}, s_{2} > 0$, respectively, satisfying (36), and also let $q_{i} >0$ $(i=1, 2, \ldots, n)$.

- If $\frac{(i+t_{2})^{s_{2}}}{(i+1+t_{2})^{s_{2}}} \leq \frac{q_{i+1}}{q_{i}}$ $(i=1, \ldots, n)$ and the base of log is greater than 1, then
  43 $$ \sum_{i=1}^{n} q_{i} \log \biggl(\frac{1}{q_{i} (i+t_{2})^{s_{2}} H_{n, t_{2}, s_{2}}} \biggr) \geq H(\mathbf{q}). $$
  If the base of log is in between 0 and 1, then the reverse inequality holds in (43).
- If $\frac{(i+t_{1})^{s_{1}}}{(i+1+t_{1})^{s_{1}}} \geq \frac{q_{i+1}}{q_{i}}$ $(i=1, \ldots, n)$ and the base of log is greater than 1, then
  44 $$ H(\mathbf{q}) \leq \sum_{i=1}^{n} q_{i} \log \biggl(\frac{1}{q_{i} (i+t_{1})^{s_{1}} H_{n, t_{1}, s_{1}}} \biggr). $$
  If the base of log is in between 0 and 1, then the reverse inequality holds in (44).

### Proof

(a) We can prove (43), by a similar method to that given in Application 1, with substitutions $p_{i}:= 1$ and $r_{i}:= \frac {1}{(i+t_{2})^{s_{2}}H_{n, t_{2}, s_{2}}}$, in Corollary 1(a) instead of Corollary 4(a).

(b) For this part, switch the role of **p** and **r** in part (a), like $p_{i}:= \frac{1}{(i+t_{1})^{s_{1}}H_{n, t_{1}, s_{1}}}$ and $r_{i}:= 1$ $(i=1,2,\ldots, n)$, and applying Corollary 1(b) instead of Corollary 4(b), we get (44). □

At the end, in the following application, we use three of the Zipf-Mandelbrot laws for different parameters.

### Application 6

Assume **p**, **q** and **r** to be the Zipf-Mandelbrot laws with parameters $n \in\{1,2, \ldots\}$, $t_{1}, t_{2}, t_{3}\geq0$ and $s_{1}, s_{2}, s_{3}> 0$, respectively, satisfying (36).

- If $\frac{(i+1+t_{2})^{s_{2}}}{(i+1+t_{3})^{s_{3}}} \leq \frac{(i+t_{2})^{s_{2}}}{(i+t_{3})^{s_{3}}}$ $(i=1, \ldots, n)$ and the base of log is greater than 1, then
  45 $$\begin{aligned} &\sum_{i=1}^{n} \frac{1}{(i+t_{3})^{s_{3}}H_{n, t_{3}, s_{3}}} \log \biggl(\frac{(i+t_{2})^{s_{2}}H_{n, t_{2}, s_{2}}}{(i+t_{3})^{s_{3}}H_{n, t_{3}, s_{3}}} \biggr) \\ &\quad \leq \sum_{i=1}^{n} \frac{1}{(i+t_{1})^{s_{1}}H_{n, t_{1}, s_{1}}} \log \biggl(\frac{(i+t_{2})^{s_{2}}H_{n, t_{2}, s_{2}}}{(i+t_{1})^{s_{1}}H_{n, t_{1}, s_{1}}} \biggr). \end{aligned}$$
  If the base of log is in between 0 and 1, then the reverse inequality holds in (45).
- If $\frac{(i+1+t_{2})^{s_{2}}}{(i+1+t_{3})^{s_{3}}} \geq \frac{(i+t_{2})^{s_{2}}}{(i+t_{3})^{s_{3}}}$ $(i=1, \ldots, n)$ and the base of log is greater than 1, then
  46 $$\begin{aligned} &\sum_{i=1}^{n} \frac{1}{(i+t_{3})^{s_{3}}H_{n, t_{3}, s_{3}}} \log \biggl(\frac{(i+t_{2})^{s_{2}}H_{n, t_{2}, s_{2}}}{(i+t_{3})^{s_{3}}H_{n, t_{3}, s_{3}}} \biggr) \\ &\quad \geq \sum_{i=1}^{n} \frac{1}{(i+t_{1})^{s_{1}}H_{n, t_{1}, s_{1}}} \log \biggl(\frac{(i+t_{2})^{s_{2}}H_{n, t_{2}, s_{2}}}{(i+t_{1})^{s_{1}}H_{n, t_{1}, s_{1}}} \biggr). \end{aligned}$$
  If the base of log is in between 0 and 1, then the reverse inequality holds in (46).

### Proof

(a) Let $p_{i}:= \frac{1}{(i+t_{1})^{s_{1}} H_{n, t_{1}, s_{1}}}$, $q_{i}:= \frac{1}{(i+t_{2})^{s_{2}} H_{n, t_{2}, s_{2}}}$ and $r_{i}:= \frac{1}{(i+t_{3})^{s_{3}} H_{n, t_{3}, s_{3}}}$. Here $p_{i},q_{i}$ and $r_{i}$ are decreasing over $i=1, \ldots, n$. Now, we investigate the behavior of $\frac{\mathbf{r}}{\mathbf{q}}$.

Take

$$\begin{aligned} &\frac{r_{i}}{q_{i}}= \frac{(i+t_{2})^{s_{2}} H_{n, t_{2}, s_{2}}}{(i+t_{3})^{s_{3}} H_{n, t_{3}, s_{3}}} \quad \mbox{and}\quad \frac{r_{i+1}}{q_{i+1}}= \frac {(i+1+t_{2})^{s_{2}} H_{n, t_{2}, s_{2}}}{(i+1+t_{3})^{s_{3}} H_{n, t_{3}, s_{3}}}, \\ &\frac{r_{i+1}}{q_{i+1}} - \frac{r_{i}}{q_{i}} = \frac {(i+1+t_{2})^{s_{2}} H_{n, t_{2}, s_{2}}}{(i+1+t_{3})^{s_{3}} H_{n, t_{3}, s_{3}}} - \frac{(i+t_{2})^{s_{2}} H_{n, t_{2}, s_{2}}}{(i+t_{3})^{s_{3}} H_{n, t_{3}, s_{3}}}, \\ &\frac{r_{i+1}}{q_{i+1}} - \frac{r_{i}}{q_{i}} = \frac{H_{n, t_{2}, s_{2}}}{H_{n, t_{3}, s_{3}}} \biggl[ \frac {(i+1+t_{2})^{s_{2}}}{(i+1+t_{3})^{s_{3}}} - \frac {(i+t_{2})^{s_{2}}}{(i+t_{3})^{s_{3}}} \biggr]; \end{aligned}$$

the R.H.S. is non-positive by using the assumption, which shows that $\frac{\mathbf{r}}{\mathbf{q}}$ is decreasing, therefore using Corollary 4(a) we get (45).

(b) If we replace $\frac{\mathbf{r}}{\mathbf{q}}$ with $\frac {\mathbf{p}}{\mathbf{q}}$ in part (a) and use Corollary 4(b), we get (46). □

## Conclusions

In this paper we show how the Shannon entropy is connected to the theory of majorization. They are both linked to the measure of disorder in a system. However, the theory of majorization usually gives stronger criteria than the entropic inequalities. The theory of majorization and the notion of entropic measure of disorder are closely related. Based on this fact, the aim of this paper is to look for majorization relations with entropic inequalities. We give some generalized results for Csiszár *f*-divergence of majorization inequality. We mention two special cases of these generalized results; the first case corresponds to the entropy of a discrete probability distribution, and the second case corresponds to the relative entropy or the Kullback-Leibler distance between two probability distributions. The cumulative distribution function (CDF) is an important application of majorization. We give several applications by using the Zipf-Mandelbrot law with (CDF).
